# Assessing water permeability of aquaporins in a proteoliposome-based stopped-flow setup

**DOI:** 10.1016/j.xpro.2022.101312

**Published:** 2022-04-13

**Authors:** Jonas Hyld Steffen, Julie Winkel Missel, Tamim Al-Jubair, Philip Kitchen, Mootaz M. Salman, Roslyn M. Bill, Susanna Törnroth-Horsefield, Pontus Gourdon

**Affiliations:** 1 Department of Biomedical Sciences, University of Copenhagen, 2200 Copenhagen, Denmark; 2 Department of Biochemistry and Structural Biology, Lund University, P.O. Box 124, 221 00 Lund, Sweden; 3 College of Health and Life Sciences, Aston University, Aston Triangle, B4 7ET Birmingham, UK; 4 Department of Physiology, Anatomy and Genetics, Kavli Institute for NanoScience Discovery, University of Oxford, Parks Road, OX1 3PT Oxford, UK; 5 Oxford Parkinson’s Disease Centre, University of Oxford, Oxford, UK; 6 Department of Experimental Medical Science, Lund University, P.O. Box 118, 221 00 Lund, Sweden

**Keywords:** Single-molecule Assays, Cell Membrane, Protein Biochemistry

## Abstract

Aquaporins (AQPs) are water channels embedded in the cell membrane that are critical in maintaining water homeostasis. We describe a protocol for determining the water permeation capacity of AQPs reconstituted into proteoliposomes. Using a stopped-flow setup, AQP embedded in proteoliposomes are exposed to an osmogenic gradient that triggers water flux. The consequent effects on proteoliposome size can be tracked using the fluorescence of an internalized fluorophore. This enables controlled characterization of water flux by AQPs.

For complete details on the use and execution of this protocol, please refer to Kitchen et al. (2020).

## Highlights

• Purified human AQP is encapsulated in proteoliposomes with fluorophore
• Quantification of aquaporin (AQP) water permeability using a proteoliposome assay
• Using stopped-flow, an osmogenic gradient triggers water flux through AQP

## Graphical abstract

**Figure:**
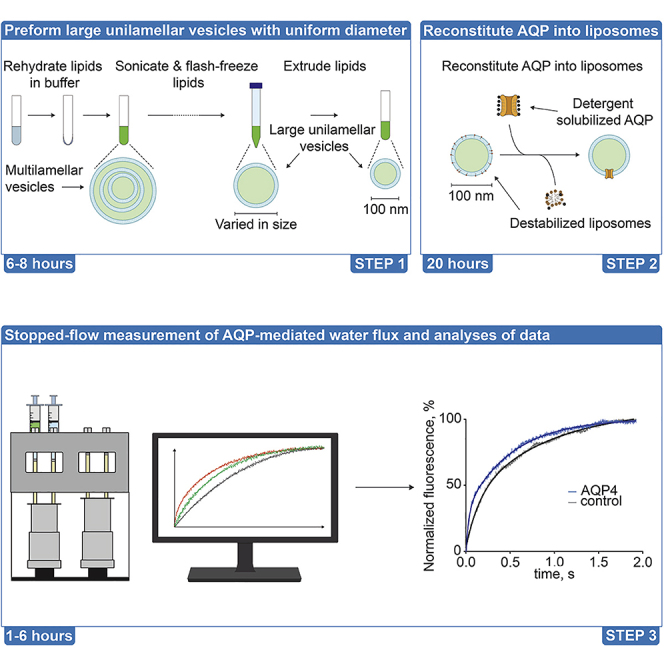

## Teaser

Aquaporins (AQPs) are water channels embedded in the cell membrane that are critical in maintaining water homeostasis. We describe a protocol for determining the water permeation capacity of AQPs reconstituted into proteoliposomes. Using a stopped-flow setup, AQP embedded in proteoliposomes are exposed to an osmogenic gradient that triggers water flux. The consequent effects on proteoliposome size can be tracked using the fluorescence of an internalized fluorophore. This enables the controlled characterization of water flux by AQPs.

## Before you begin

For this protocol, purified aquaporin is needed such as human aquaporin 4 (AQP4). An example of how to purify human AQPs, specifically AQP4 using *Pichia pastoris,* is provided in Kitchen et al. (2020).

### Protocol overview

To assess whether the isolated AQP is functionally active, the protein can be reconstituted into large unilamellar vesicles (LUVs) to form proteoliposomes. The water permeation capacity of the purified AQP can then be characterized through studies of water fluctuations across the liposomes. In this protocol we outline reconstitution of AQP4 into liposomes with an internalized fluorophore and we describe how to measure water flux using a stopped-flow setup. This setup represents a powerful method to investigate AQP activity and kinetics, as parameters such as the osmogene, pH etc. can be systematically investigated.

AQP4 is a water channel that is natively expressed in the membrane of tissues such as the collecting duct of kidney cells (Frigeri et al., 1995), parietal cells in the stomach (Misaka et al., 1996) and is abundantly expressed in CNS astrocytes (Oshio et al., 2004). The lipid composition of astrocytes is high in phospholipids such as phosphatidylcholines, cholesterol, and sphingomyelin (Fitzner et al., 2020). Therefore, in this protocol a mixture of 1-palmitoyl-2-oleoyl-sn-glycero-3-phosphocholine (POPC, Sigma - 42773), 2-oleoyl-1-palmitoyl-sn-glycero-3-phospho-rac-(1-glycerol) (POPG, Sigma - 76559) and cholesterol (Sigma - C8667) in a 2:1:2 ratio (w/w/w) is used. POPG is included to help stabilize the LUV formation and minimize aggregation, as previously reported (Tong et al., 2012).

The composition of the liposomes changes the mechanical and structural properties of the bilayer, such as its elastic properties and hydrocarbon thickness. This in turn changes the unit permeabilities for AQP4 (Tong et al., 2012).

For other AQPs, bacterial lipids such as *E. coli* polar lipid extracts have also been used for successful reconstitution of e.g., AQP1 (Walz et al., 1994), AQP2 (Werten et al., 2001), AQP8 (Liu et al., 2006) and AQP10 (Hering et al., 2020; Missel et al., 2021). Thus, this protocol can easily be adapted for many different AQPs.

## Key resources table

**Table:** 

REAGENT or RESOURCE	SOURCE	IDENTIFIER
**Chemicals, peptides, and recombinant proteins**		

1-palmitoyl-2-oleoyl-sn-glycero-3-phosphocholine (POPC)	Sigma-Aldrich	Cat# 42773
2-oleoyl-1-palmitoyl-sn-glycero-3-phospho-rac-(1-glycerol) (POPG)	Sigma-Aldrich	Cat# 76559
5 (6)-carboxyfluorescein	Sigma-Aldrich	Cat# 21877
Cholesterol	Sigma-Aldrich	Cat# C8667
Chloroform	Sigma-Aldrich	Cat# 372978
Glycerol	VWR	Cat# 24388.320
HEPES sodium salt	AppliChem	Cat# A1069,1000
NaCl	VWR	Cat# 27810.364
n-Octyl glucoside, Anagrade	Anatrace	Cat# O311HA 25 GM
Sucrose	Sigma-Aldrich	Cat# S7903-1KG
Triton-X-100	VWR	Cat# M143-1L

**Software and algorithms**		

GraphPad Prism Version 9	GraphPad Prism Software	https://www.graphpad.com/support/faq/prism-900-release-notes/
Pro-Data Viewer	Applied Photosystems	https://www.photophysics.com/support-and-service/documents-and-software/pro-data-sx-software-update

**Other**		

1.5 mL Eppendorf tube	Nerbe-plus	Cat# 04-212-1200
15 mL Falcon tube	Labsolute	Cat# 7696714
50 mL round bottom flask	Glassco	Cat# 52-1485
Bath sonicator	Bandelin Electronic	Cat# 301
Dialysis tube, 12–14 kDa MWCO	Spectrum	Cat# 132700
Fiberlite™ F23-48 × 1.5 Fixed-Angle Rotor	Thermo Scientific	Cat# 096-484075
Glass vial	Shimadzu	Cat# 228-25315-91
Mini-extruder	Avanti Lipids	Cat# 610000
SX-20 Stopped-Flow Spectrometer	Applied Photosystems	https://www.photophysics.com/systems/stopped-flow-spectrometry/sx20-stopped-flow/system-information/
Whatman Nuclepore (0.1 μm)	Whatman	Cat# 800309
Whatman drain disc	Whatman	Cat# 230300

## Materials and equipment

***Alternatives:*** In the key resources table, we have listed the instruments and models used in our laboratory. However, these specific models are not crucial for the success of the protocol.

***Note:*** Stock solutions of 5 M NaCl, 20 mM 5 (6)-carboxyfluorescein and 80% glycerol can be filtered through 0.22 μm filters and stored at 20°C–22°C for 5–6 months. Stock solutions of 1 M HEPES pH 8 can be stored at 4°C for 1–2 months after being sterile filtered through a 0.22 μm filter.

**Table:** Lipid-resuspension-buffer

Reagent	Final concentration	Amount
1 M HEPES pH 8.0 stock	20 mM	200 μL
5 M NaCl stock	200 mM	400 μL
20 mM 5 (6)-carboxyfluorescein stock	10 mM	5 mL
Deionized H_2_O	n/a	up to 10 mL
**Total**		**10 mL**

Use freshly made solution.

**Table:** Reconstitution-buffer. Use the same detergent as in the SEC buffer that is used to purify the protein of interest.

Reagent	Final concentration	Amount
1 M HEPES pH 8.0 stock	20 mM	200 μL
5 M NaCl stock	200 mM	400 μL
Glycerol, 80% stock	25% v/v	3,125 μL
n-Octyl glucoside	1% w/v	100 mg
Deionized H_2_O	n/a	up to 10 mL
**Total**		**10 mL**

Use freshly made solution.

**Table:** Dialysis-buffer

Reagent	Final concentration	Amount
1 M HEPES pH 8.0 stock	20 mM	20 mL
5 M NaCl stock	200 mM	40 mL
Deionized H_2_O	n/a	up to 1,000 mL
**Total**		**1,000 mL**

Use freshly made solution.

**Table:** Reaction-buffer

Reagent	Final concentration	Amount
1 M HEPES pH 8.0 stock	20 mM	200 μL
5 M NaCl stock	200 mM	400 μL
Sucrose	200 mM	685 mg
Deionized H_2_O	n/a	up to 10 mL
**Total**		**10 mL**

Use freshly made solution.

## Step-by-step method details

### Prepare preformed liposomes

**Timing: 8 h**

This step describes the preparation of preformed liposomes from a well-defined ratio of pure lipids.

Dissolve the required lipids (POPC:POPG:Cholesterol in a ratio of 2:1:2) in chloroform (Sigma, 372978) to a concentration of 25 mg/mL in a glass vial.

**CRITICAL:** It is important to use glassware for handling and storing lipids that are dissolved in chloroform, as this will prevent contamination of polymers from the plastics (Pidgeon et al., 1989). Furthermore, as chloroform is volatile, pipetting exact volumes can be difficult, so supplementation of chloroform should be performed rapidly.

1. Dehydrate the sample using a weak nitrogen stream while hand rotating the glass vial (4 mL test tube, Shimadzu) until all liquid is removed, leaving a lipid film on the bottom.

***Note:*** After dissolving the lipids in chloroform in the glass vials, it can be an advantage to mix and dehydrate them in a round-bottomed flask (50 mL round-bottomed flask, Glassco) while also rotating the flask. By doing so it will be easier to dry the lipids in a thin film, for subsequent rehydration.

2. Continue dehydrating the thin lipid film for 2–4 h or overnight in a vacuum desiccator for complete removal of the chloroform.

***Note:*** If no desiccator is available, the glass vial can be placed in a 50 mL Falcon tube, the nitrogen outlet left inside the tube, finally wrapping the tube with parafilm. If dehydrating in a round-bottomed flask, the nitrogen outlet can be placed in the flask with parafilm wrapped around the neck. Perforate the parafilm by pinching small holes to ensure that excess nitrogen can flow out. The perforation should be sufficient to permit that excess nitrogen can leave the container.

**CRITICAL:** It is important to rehydrate the lipids immediately. In their dried state lipids are sensitive to oxidation. Thus, proceed with the next steps immediately following the dehydration.

3. Rehydrate the lipid film in lipid-resuspension buffer to a final concentration of 20 mg/mL lipid in the solution.
  a. Work swiftly to avoid lipid oxidation and/or keep the vial covered.
  b. Ensure that the suspension is homogenous through gently stirring or tapping the glass vial. In this way multilamellar vesicles (MLVs) will spontaneously form (Bangham et al., 1965).

***Note:*** The fluorophore 5(6)-carboxyfluorescein is orange red as a powder as well as in a liquid stock (20 mM) due to self-quenching. The color of the fluorophore will change during the process of preparing proteoliposomes as the fluorophore will be diluted and will emit a neon-green color. Upon removal of excess fluorophore after dialysis and centrifugation, the suspension will be colorless.

**Figure 1:**
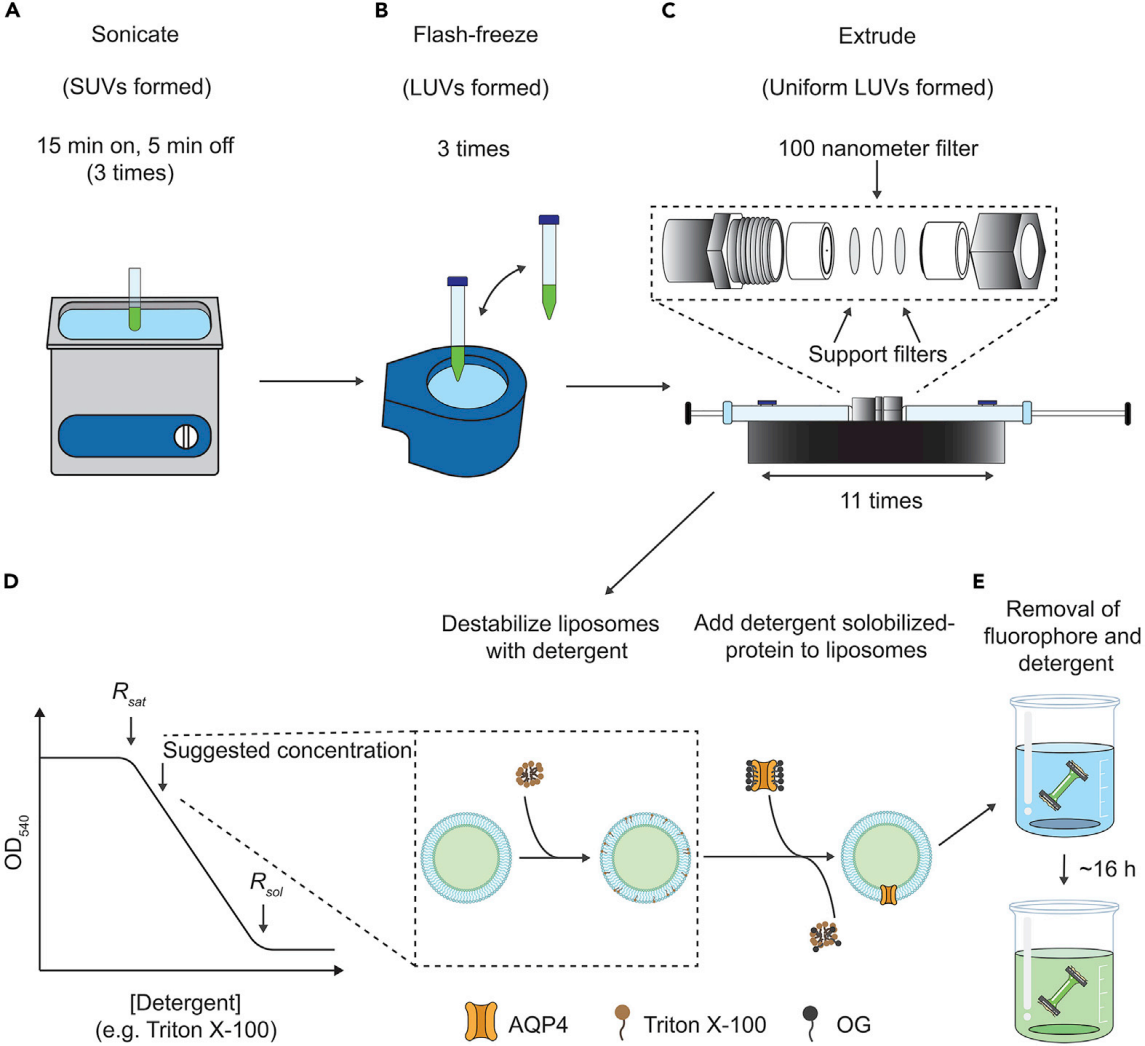
Schematic overview of preparation of AQP4 containing proteoliposomes (A) Following dehydration of the lipids to remove chloroform, the lipids are rehydrated in lipid resuspension buffer with fluorophore. Subsequently, the lipid suspension is sonicated to form small unilamellar vesicles (SUVs). (B) The lipid suspension is then flash frozen in liquid nitrogen and the suspension slowly thawed at 20°C–22°C three times to form large unilamellar vesicles (LUVs). (C) The sample is extruded 11 times using a 100 nm polycarbonate filter to form LUVs with a uniform size (diameter of 100 nm). (D) The lipid sample is titrated with detergent to determine *R*_*sat*_ and *R*_*sol*_ by measuring the optical density at 540 nm (Geertsma et al., 2008). *R*_*sat*_ and *R*_*sol*_ refer to the detergent concentration at which the membrane is saturated with detergent and fully solubilized, respectively. The middle arrow indicates the suggested detergent concentration at which the liposomes can be combined with the detergent-purified AQP for reconstitution. (E) Removal of excess fluorophore from the external side of the liposome as well as detergent to seal the proteoliposomes. Panel D is adapted from (Rigaud and Levy, 2003).

4. Sonicate the lipid suspension in three cycles (15 min on / 5 min off) by placing the glass vial in a bath sonicator at 35 kHz (Sonorex RK100, Bandelin Electronic) or the equivalent (Figure 1A).
  a. Cover the glass vial with e.g., parafilm. In this step small unilamellar vesicles (SUVs) are formed.

**CRITICAL:** SUVs should not be stored above their transition temperature for more than 24 h. The transition temperature of POPC and POPG is −2°C.

5. Transfer the lipid solution to a plastic tube, while glass will break, and flash freeze the sonicated sample using liquid nitrogen, and thaw at 20°C–22°C (Figure 1B).
  a. Repeat step 5 three times. This will fuse the SUVs into LUVs with fluorophore incorporated (Pick, 1981).

**Pause point:** In our laboratory we often introduce a pause point after the rehydration step (step 3) when MLVs have been formed, by storing the lipid suspension at −80°C. However, it is also possible to store the lipid solution after sonication or after the third freezing step, also at −80°C. The formed SUVs are stable for a couple of weeks before they start to aggregate and fuse into LUVs or MLVs (Lasic, 1988), while the MLVs can be stored for longer periods.

### Reconstitute the AQP into detergent-destabilized liposomes

**Timing: 20 h**

In this step, the reconstitution of AQP4 into preformed liposomes destabilized in detergent is described.

6. Equilibrate a mini extruder (Mini-Extruder, Avanti) filters (2× support filter, Whatman – 230300) and 1× 100 nm polycarbonate filter (Whatman Nucleopore – 800309)) using reconstitution buffer. The filters are assembled with a support filter on each side of the nanometer filter (Figure 1C).

**CRITICAL:** Prewash the extruder to ensure that it is equilibrated in the desired buffer and certify that the extruder does not leak before the sample is applied.

7. Dilute the lipid suspension to 1 mL in reconstitution buffer and extrude the non-uniform LUVs 11 times through the 100 nm filter membrane to form uniform LUVs for reconstitution.
  a. The liposome suspension is initially opaque and will become transparent following the treatment (Figure 1C).

***Note:*** it can be difficult to remove the fluorophore completely from the individual parts of the extruders. Thus, if different proteins are assayed, it can be advantageous to have two sets of extruders, one for proteins that have fluorescein incorporated and one for non-fluorescein work. Residual fluorophore can also be removed by bath sonication for 10 min.

8. Dilute the LUVs 5× to reach a lipid concentration of 4 mg/mL in the reconstitution buffer.

**CRITICAL:** To stabilize the detergent-solubilized protein samples, the LUVs should be diluted in reconstitution buffer including 25% v/v glycerol to reach a final glycerol concentration of 20% v/v.

9. Titrate the LUVs with a detergent such as Triton X-100 at 20°C–22°C until the liposomes are saturated with detergent and the so-called *R*_*sat*_ is reached.
  a. Supplement aliquots of 5 μL 10% v/v Triton X-100 and mix the suspension gently.
  b. Let the Triton X-100/LUV suspension equilibrate shortly (approx. 20–30 s) between each addition.
  c. The destabilization of the liposomes can be monitored through assessment of the optical density of the lipid suspension at 540 nm (Figure 1D). In our hands, *R*_*sat*_ is typically reached at a 0.02% w/v Triton X-100 concentration.

**CRITICAL:** The appropriate concentration of detergent (e.g., Triton X-100) to destabilize the liposomes needs to be determined empirically through titration. At detergent concentrations below *R*_*sat*_*,* the efficiency of protein insertion is low, while at detergent concentrations somewhat above *R*_*sat*_*,* insertion is typically efficient (Geertsma et al., 2008). At elevated detergent concentrations, the liposomes will start to disintegrate forming lipid-detergent mixed micelles, rendering the solution optically transparent (*R*_*sol*_) (Rigaud and Levy, 2003).

10. Divide the liposomes into the required number of tubes, with 500 μL solution (or 2 mg lipids) in each.
11. Supplement the purified AQP to the detergent destabilized liposomes in the desired lipid-to-protein-ratio (LPR, w/w) (Figure 1D).
  a. For AQP4, a LPR of 200 is prepared by adding 10 μL of 1 mg/mL protein to the solution. The LPR may need to be optimized for each target.

**CRITICAL:** Include a sample with empty liposomes that has been treated with Triton X-100, as this will serve as a negative control in the following activity measurements. Also, do at least triplicates of the reconstitution procedure for statistical purposes when quantifying the water permeability (see below). Preferably the triplicates should represent three different biological samples (purifications) but triplicates using the same purification will assess differences in reconstitution effectiveness.

12. Mix by gently inverting the tube 2–3 times and incubate for 1 h at 4°C.
13. Dialyze the sample against 1 L dialysis buffer (see above) using a 12–14 kDa cut-off membrane (Spectrum, 132700) for 16 h at 4°C (Figure 1E).
  a. This step is critical to reduce the detergent concentration of the mixture and to remove residual fluorophore from the external side of the liposomes.
14. Centrifuge at 57,000 × *g* for 1.5 h in a small ultra-rotor (Thermo Scientific - F23-48 × 1.5) and remove the supernatant.
15. Resuspend the lipidic pellets of three independent reconstitutions (triplicates) in 1 mL dialysis buffer (see above).

### Assay water flux through proteoliposomes by stopped-flow spectrometry

**Timing: 1–6 h (depending on the number of constructs and conditions measured)**

**Figure 2:**
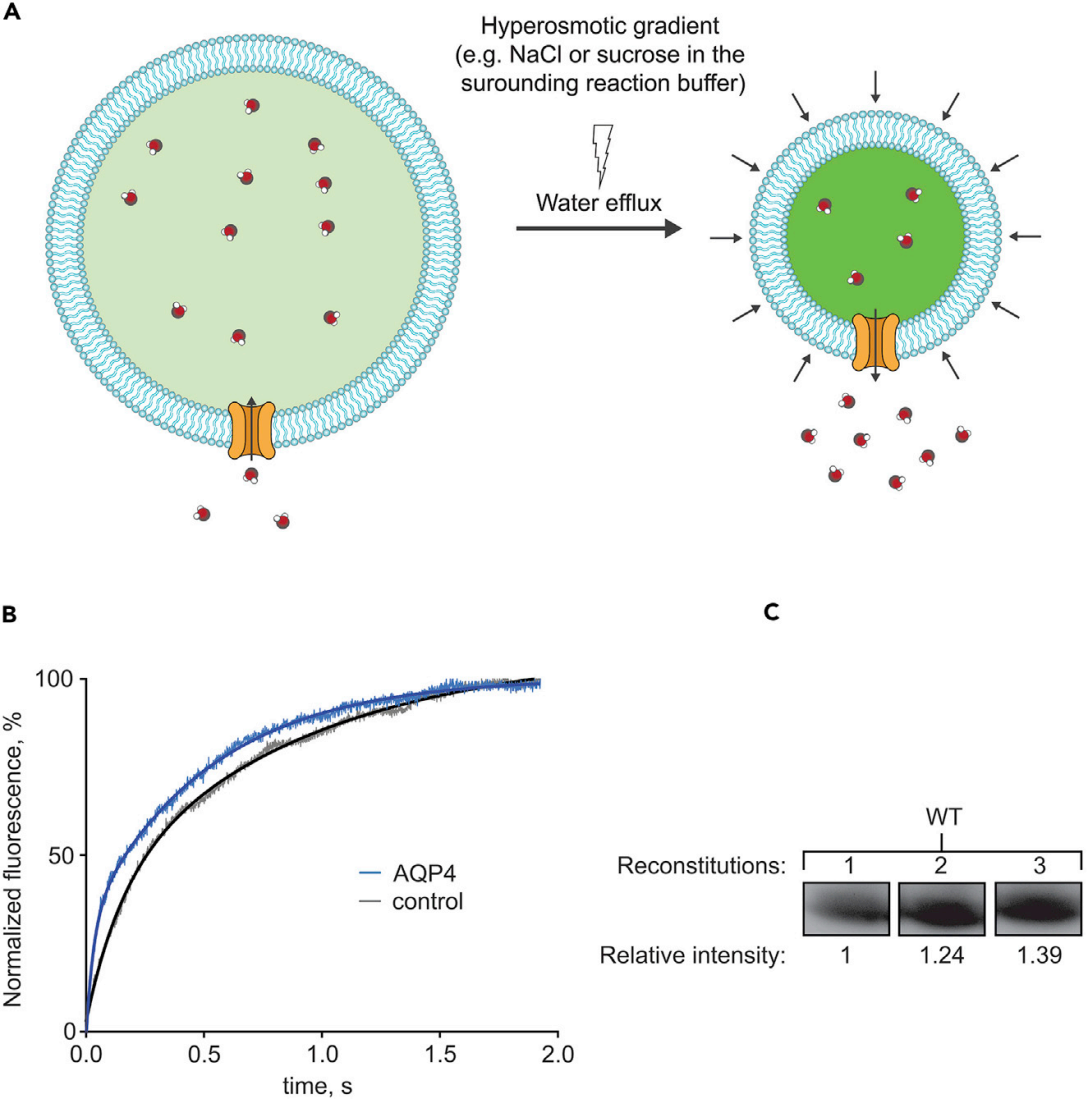
Assessing water flux capacity of isolated AQPs using stopped flow (A) Schematic overview of the proteoliposome-based assay. Proteoliposomes with AQP inserted are mixed with reaction buffer and exposed to a hyperosmotic gradient in a stopped-flow setup. This triggers water efflux and shrinkage of the proteoliposomes, leading to an increase in the light scattered by (5)6-carboxyfluorescein measured at 495 nm. (B) Representative measurement of the water permeability of reconstituted AQP4 (blue line) and control empty liposomes (black line). (C) Representative western blot of three separate reconstitutions of an AQP to scale the water transport capacity relative to the protein levels incorporated in the proteoliposomes. Quantification is performed using e.g., the software ImageJ. The efficiency can be used to adjust the *k*_1_-value of each reconstitution, and hence to adjust the *P*_*f*_-value.

***Note:*** Depending on the AQP and measurement, other osmogents such as NaCl (Missel et al., 2021) and sorbitol (Liu et al., 2006) may also be exploited.

16. Mix the proteoliposomes with reaction buffer in a stopped-flow spectrometer such as a SX-20 (Applied Photophysics) and collect data at 495 nm at a 90° angle for 2 s (or adjust the length of the measurement depending on the construct and condition).
  a. As proteoliposomes are impermeable to sucrose, the osmolytic gradient will trigger water efflux and shrinkage of the proteoliposomes and thereby an increase in light scattering by (5)6-carboxyfluorescein at this wavelength (Figure 2A).

## Expected outcomes

A representative measurement on the water permeability of AQP4 fitted to the above-mentioned double exponential equation is shown in Figure 2B. We have obtained similar data using this procedure also for other AQPs (Hering et al., 2020; Missel et al., 2021).

## Limitations

The dead-time of the stopped-flow apparatus (the time it takes between mixing and start of measurement) limits how fast reactions can be measured (for our setup it is approximately 1 ms). Thus, in certain cases it may be necessary to decrease the activity of the protein, if possible (e.g. through introduction of mutations). Furthermore, the technique described in this protocol is measuring on an isolated system, with a lipid composition that is non-native. Consequently, the protein may behave differently than in native cells. However, this is of course also the strength of this assay, as it can reveal the kinetics and transport mechanism of isolated proteins, without cellular background.

## Quantification and statistical analysis

Analyze and plot the data (e.g., using the software GraphPad Prism (GraphPad software), Pro-Data Viewer (Applied Photosystems) or MatLab (MathWorks)); each sample is averaged from 10 readings, normalized and fitted using the following double exponential equation:

$$y=y_{0}+A_{1}e^{-k_{1}\left(x-x_{0}\right)}+A_{2}e^{-k_{2}\left(x-x_{0}\right)}$$

where *A*_1_ and *A*_2_ are the respective amplitudes and *k*_1_ and *k*_2_ are the fitted *k*-rate constants (Figure 2B). The larger of the *k*-rate constants (*k*_1_) represents the *k*-rate of the embedded AQP4, while the smaller *k*-rate constant (*k*_2_) is unaffected by the changes in the reconstitution efficiency. Thus, *k*_1_ is used to calculate the osmotic water permeability, *P*_*f*_ using the following equation (Tong et al., 2012):

$$P_{f}\left(cm/s\right)=\frac{k}{\left(\frac{S}{V_{0}}\right)*V_{w}*\left(C_{out}-C_{in}\right)}$$

where (*S*/*V*_0_) is the initial surface area to volume ratio of the liposome, *V*_*w*_ is the partial molar volume of water (18 *cm*^3^/*mol*) and *C*_*in*_ and *C*_*out*_ are the initial concentrations of solute on the inside and on the outside of the vesicles, respectively.

For statistical purposes, each reconstitution should be assessed at least as triplicates (e.g., three separate reconstitutions). Consequently, samples from step 15 above should be assessed using SDS-PAGE and/or western blot, to quantify the amount of incorporated protein using e.g., the software ImageJ (NIH) (Schneider et al., 2012) (Figure 2C). Use this to adjust the *k*_1_-value of each reconstitution to adjust the *P*_*f*_-value. The value may vary between mutant forms.

## Troubleshooting

### Problem 1

The lipid solution will not go through 100 nm filter in the extruder (step 7).

### Potential solution

Use a filter with a larger pore size (e.g., 200 nm Whatman Nucleopore – 10417004, or 400 nm Whatman Nucleopore – 10417104) as an initial reduction of liposome. Then, subsequently extrude through a 100 nm pore size filter.

### Problem 2

The protein of interest is not inserted into the liposomes (step 11).

### Potential solution

Following supplementation of detergent solubilized AQP to the destabilized liposomes, it is important to remove detergent to incorporate the protein into the liposomes. This can be achieved through dilution, dialysis, SEC, or polystyrene beads (Bio-Beads SM2, Bio-Rad). We have not exploited Amberlite XAD2 or cyclodextrin, but these strategies may also be used for the purpose of removing the detergent. In our protocol, we employ dialysis to remove the detergent, as this method typically removes detergents with a high critical micelle concentration (such as *n*-octyl-β-D-glucoside) efficiently (Geertsma et al., 2008) (step 13). In case the dialysis does not work, one of the other methods can be attempted. Detergents with a low critical micelle concentration (such as dodecyl-β-D-maltopyranoside) will require extended dialysis periods, and hence Bio-Beads or SEC may represent more suitable solutions.

Furthermore, a stable and pure protein sample is required to achieve efficient incorporation into the liposome. It is therefore recommended to perform a SEC purification of the target protein immediately prior to reconstitution to have a sample as fresh as possible.

### Problem 3

The reaction is too fast or slow for the stopped-flow machine to read a steady signal (step 16).

### Potential solution

Decrease the activity of the protein so that the reaction takes place more slowly. This can be achieved through optimization of several different parameters, such as the lipid composition (step 1), the LPR (step 11) and the osmotic gradient (step 16). For AQP4, cholesterol is added to the lipid mixture when preparing the liposomes to make the membrane more rigid, and thereby decreasing water flux. The LPR is also increased relative to other AQPs (Hering et al., 2020; Missel et al., 2021) which will further slowdown the water transport since fewer proteins are present in the liposomes. The reverse, with a too slow process, can also happen, in which case the parameters need to be optimized accordingly.

## Resource availability

### Lead contact

Further information and requests for resources and reagents should be directed to and will be fulfilled by the lead contact, Pontus Gourdon Email: pontus.gourdon@med.lu.se.

### Materials availability

This study did not generate new unique reagents.

## Data Availability

This study did not generate/analyze datasets and codes.
